# Early changes in pulmonary functions after mitral valve replacement

**DOI:** 10.4103/1817-1737.33699

**Published:** 2007

**Authors:** Pankaj Saxena, Suvitesh Luthra, Rajinder Singh Dhaliwal, Surinder Singh Rana, Digambar Behera

**Affiliations:** Department of Cardiovascular and Thoracic Surgery, Postgraduate Institute of Medical Education and Research, Chandigarh, India; * *Department of Pulmonary Medicine, Postgraduate Institute of Medical Education and Research, Chandigarh, India*

**Keywords:** Mitral valve replacement, pulmonary function test

## Abstract

**BACKGROUND:**

This study evaluates changes in pulmonary functions before and after mitral valve replacement (MVR).

**MATERIALS AND METHODS:**

Twenty-five patients with rheumatic mitral lesions who had undergone MVR were divided into three groups, based on New York Heart Association (NYHA) class. They were evaluated for changes in pulmonary functions, preoperatively and postoperatively at 1 week, 1 month and 3 months to find any improvements after MVR.

**RESULTS:**

Forced vital capacity (FVC), forced expiratory volume in 1 second (FEV1), peak expiratory flow rates were universally found to be decreased preoperatively. Total lung capacity (TLC) and diffusion capacity (DLCO) were significantly reduced preoperatively in NYHA Class III and IV. The pulmonary functions further declined at 1 week after surgery. Except for FVC in NYHA Class IV (32.3% improvement, *P* < 0.05), the changes were statistically insignificant.

**CONCLUSIONS:**

Pulmonary functions deteriorate immediately after surgery and then recover gradually over a period of 3 months. However, they remain below the predicted values.

Mitral valve disease has important effects on the pulmonary vasculature. Pulmonary hypertension in these patients usually results from a combination of transmission of raised left atrial pressure, pulmonary arteriolar constriction and organic obliterative changes in the pulmonary vasculature.

Pulmonary dysfunction is attributed to interstitial and alveolar edema, reactive fibrosis, previous pulmonary infarctions, pleural effusion and decreased lung volumes with compressive atelectasis. These changes cause a marked reduction in lung compliance, an increase in the work of breathing and a redistribution of pulmonary blood flow from the bases to apices.[1]

Pulmonary physiology and mechanics are further disturbed after thoracic surgery. A restrictive pattern develops, which may persist for weeks to months postoperatively.[2] Measurement of lung volumes reveals changes in total lung capacity, vital capacity, residual volume and functional residual capacity. The reduction of these lung volumes leads to reduction in the available surface area for blood-gas exchange. As functional residual capacity is reduced up to 50%, it approaches closing volume, predisposing to atelectasis.[3]

Additional risks for pulmonary dysfunction associated with open heart surgery include the use of ice slush for topical cooling and cardiopulmonary bypass itself resulting into phrenic nerve injury and diaphragmatic dysfunction with atelectasis and a restrictive ventilatory pattern.[4] Cardioplegia may passively enter the pulmonary circulation and damage the endothelial cells in the lungs due to high potassium concentration and result in atelectasis.[5–7]

The present study was conducted to document the changes in pulmonary functions in mitral disease and study the reversibility and patterns of improvement after surgery.

## Materials and Methods

The study was conducted in the Departments of Cardiothoracic Surgery and Pulmonary Medicine at Nehru Hospital of Postgraduate Institute of Medical Education and Research, Chandigarh. The hospital review board approved the study, and informed consent was obtained from all the patients.

Twenty-five patients (male/female - 10/15) suffering from mitral valve disease requiring surgical intervention were studied. The patients were subdivided on the basis of New York Heart Association (NYHA) Class: There were 8 patients in NYHA Class II (M/F - 2/6), 11 patients in NYHA Class III (M/F - 6/5) and 6 patients in NYHA Class IV (M/F - 2/4).

Preoperative assessment of the cardiac lesion was done using echocardiography. Mitral valve disease was rheumatic in all the cases (mitral stenosis - 7 patients, mitral regurgitation - 3 patients and mixed lesion in 15 patients). Varying degrees of tricuspid regurgitation were present in 17 patients (68%). Seven patients (28%) had associated mild aortic valve disease, which did not require any surgical correction.

Pulmonary function tests (PFT) were done preoperatively (within 1 week before surgery) and postoperatively at 1 week, 1 month and 3 months after surgery. The following parameters were evaluated: forced vital capacity (FVC), forced expiratory volume in the first second (FEV1), FEV1/FVC ratio, peak expiratory flow rate (PEFR), residual volume (RV), total lung capacity (TLC) and diffusion capacity (DLCO). The preoperative values were analyzed according to the NYHA Class. of the patients. Criteria for interpreting pulmonary functions were based upon the recommendations of American Thoracic Society.[8]

Mitral valve replacement (MVR) was performed using median sternotomy in all the cases. Standard cardiopulmonary bypass was instituted after heparinization using aortic and bicaval canulation. Antegrade cold blood and terminal warm blood cardioplegia was used in all cases. Topical and systemic hypothermia to 30°C was employed for myocardial protection. Mitral valve was found to be thickened and fibrotic in all the cases. Valve leaflets were calcified to variable degree in 11 cases. Fourteen patients had varying severity of subvalvular pathology. One patient had left atrial clot. In none of the cases was the valve deemed to be suitable for repair. Mitral valve replacement was performed using mechanical valves in all the cases (Starr Edwards - 13, St Jude - 11, Sorin - 1). Concomitant tricuspid valve repair with deVega annuloplasty was done in 8 cases (32%). The pleura was not opened in any case. All patients had a pericardial and a retrosternal drain.

The patients were ventilated overnight, and all except one could be extubated on the first postoperative day. A strict regime of chest physiotherapy, bronchodilators and pain control was initiated after extubation. Early mobilization was encouraged. All patients were started on warfarin from the first postoperative day to maintain an international normalized ratio (INR of 2–3). Drains were removed by the second postoperative day in all patients.

FVC, FEV1, FEV/FVC, PEFR were estimated by spirometry *(Spiroanalyser ST- 200/ Fukuda Sangyo).* RV, TLC and DLCO were assessed using carbon monoxide single-breath technique using diffusion test machine *[PK Morgan (UK) Ltd.)].*

Lung volumes and diffusion capacity were compared with the standard values provided with diffusion test machine. The spirometric values were compared with those already established for the local population.[9] The postoperative pulmonary functions were compared with the preoperative values and analyzed according to the functional class. The values within the same class were analyzed for statistical significance using paired ‘t’ test. The significance of difference between the classes was analyzed using analysis of variance test.

## Results

Twenty-five patients (male/female - 10/15) suffering from mitral valve disease requiring surgical intervention were studied (male - mean age: 29.70 ± 9.45 years; range: 18-45 years; female - mean age: 32.33 ± 10.28 years; range: 14-50 years).

Patients with renal and hepatic dysfunction, coronary artery disease and emergency surgery were excluded. Duration of symptoms ranged from 1-30 years (mean duration of 7.84 years). Dyspnea was the presenting symptom in all the cases. Other symptoms included palpitation (80%), chest pain (24%), recurrent chest infections (20%), hemoptysis (8%), cerebrovascular accident (8%) and congestive heart failure (8%). Three patients had previous closed mitral valvotomy (CMV) and one patient had undergone balloon mitral valvotomy in the past. A past history of rheumatic fever during childhood was present in 12 patients (48%). All females were nonsmokers. Three males were smokers who stopped smoking after they developed significant symptoms.

Table 1 compares the preoperative values to those predicted. The changes in pulmonary functions are compared to their preoperative values in Tables 2–4. (The postoperative ‘p’ values correspond to comparisons with the preoperative value for each NYHA class).

**Table 1:** Effect of mitral disease on pulmonary function test - preoperative profile

	Predicted	Preoperative	% change	*P*-value
Class II (n=8)				
FVOφ	3.12 ± 0.41	2.10 ± 0.57	-32.7	<0.01
FEV1φ	2.63 ± 0.31	1.84 ± 0.50	-30.0	<0.05
PEFR§	362.1 3 ± 50.49	241.38 ± 97.21	-33.4	<0.05
RVφ	1.28 ± 0.1 9	1.68 ± 0.68	+31.2	>0.05
TLCφ	4.22 ± 0.58	4.14 ± 1.31	-1.9	>0.05
DLCOψ	28.1 3 ± 7.28	19.92 ± 6.28	-29.2	>0.05
Class III (n=11)				
FVCφ	3.55 ± 0.84	2.75 ± 0.95	-22.5	<0.01
FEV1φ	2.93 ± 0.71	2.55 ± 0.84	-13.0	<0.05
PEFR§	41 2.09 ± 96.70	301.73 ± 89.66	-26.8	<0.01
RVφ	1.42 ± 0.24	1.40 ± 0.39	-1.4	>0.05
TLCφ	4.85 ± 0.87	3.75 ± 1.04	-22.7	<0.01
DLCOψ	27.30 ± 3.97	17.69 ± 6.86	-35.2	<0.01
Class IV (n=6)				
FVCφ	3.35 ± 0.78	2.32 ± 1.08	-30.7	<0.05
FEV1φ	2.79 ± 0.69	1.87 ± 1.04	-32.9	<0.05
PEFR§	384.1 7 ± 85.57	242.00 ± 68.25	-37.0	<0.05
RVφ	1.32 ± 0.1 2	1.63 ± 0.82	+23.5	>0.05
TLCφ	4.58 ± 0.80	3.87 ± 1.47	-15.5	>0.05
DLCOψ	27.03 ± 3.1 8	16.31 ± 3.86	-39.7	<0.01

DLCO - Diffusion capacity, FVC - Forced vital capacity, FEV1 - Forced expiratory volume in 1 second, PEFR - Peak expiratory flow rate, RV - Residual volume, TLC - Total lung capacity.

φ Values are in liters

§ Values are in liters/min

ψ Values are in ml/mm Hg/min.

**Table 2:** Changes in pulmonary function test at 1 week after mitral valve replacement

	Preoperative	Postoperative (1 week)	% change	*P*-value
Class II (n=8)				
FVCφ	2.10 ± 0.57	1.82 ± 0.64	-13.3	>0.05
FEV1φ	1.84 ± 0.50	1.26 ± 0.33	-31.5	<0.01
PEFR§	241.38 ± 97.21	148.88 ± 7.47	-38.3	<0.01
RVφ	1.68 ± 0.68	1.78 ± 0.73	+5.9	>0.05
TLCφ	4.14 ± 1.31	3.50 ± 1.20	-15.5	<0.01
DLCOψ	19.92 ± 6.28	15.85 ± 5.46	-20.4	>0.05
Class III (n=11)				
FVCφ	2.75 ± 0.95	2.1 9 ± 0.70	-20.4	<0.05
FEV1φ	2.55 ± 0.84	2.11 ± 0.71	-17.2	<0.05
PEFR§	301.73 ± 89.66	294.82 ± 87. 13	-2.6	>0.05
RVφ	1.40 ± 0.39	1.38 ± 0.34	-1.4	>0.05
TLCφ	3.75 ± 1.04	3.22 ± 0.60	-14.1	<0.05
DLCOψ	17.69 ± 6.86	14.45 ± 2.37	-18.3	>0.05
Class IV (n=6)				
FVCφ	2.32 ± 1.08	2.11 ± 0.71	-9.0	>0.05
FEV1φ	1.87 ± 1.04	1.80 ± 0.78	-3.7	>0.05
PEFR§	242.00 ± 68.25	193.00 ± 61.43	-20.24	>0.05
RVφ	1.63 ± 0.82	1.57 ± 1.00	-3.7	>0.05
TLCφ	3.87 ± 1.47	3.68 ± 1.31	-4.9	>0.05
DLCOψ	16.31 ± 3.86	14.77 ± 1.91	-8.9	>0.05

DLCO - Diffusion capacity, FVC - Forced vital capacity, FEV1 - Forced expiratory volume in 1 second, PEFR - Peak expiratory flow rate, RV - Residual volume, TLC - Total lung capacity.

φ Values are in liters

§ Values are in liters/min

ψ Values are in ml/mm Hg/min.

**Table 3:** Changes in pulmonary function test at 1 month after mitral valve replacement

	Preoperative	Postoperative (1 month)	% change	*P*-value
Class II				
FVOφ	2.10 ± 0.57	2.27 ± 0.87	+8.1	>0.05
FEV1φ	1.84 ± 0.50	1.91 ± 0.96	+3.8	>0.05
PEFR§	241.38 ± 97.21	2 10.25 ± 80.50	-12.9	>0.05
RVφ	1.68 ± 0.68	1.65 ± 0.48	-1.8	>0.05
TLCφ	4.1 4 ± 1.31	3.76 ± 1.06	-9.2	>0.05
DLCOψ	19.92 ± 6.28	20.35 ± 7.1 2	+2.1	>0.05
Class III				
FVCφ	2.75 ± 0.95	2.63 ± 0.82	-4.7	>0.05
FEV1φ	2.55 ± 0.84	2.40 ± 0.76	-5.9	>0.05
PEFR§	301.73 ± 89.66	301.27 ± 88.55	-0.1	>0.05
RVφ	1.40 ± 0.39	1.42 ± 0.42	+1.4	>0.05
TLCφ	3.75 ± 1.04	3.66 ± 0.65	-2.4	>0.05
DLCOψ	17.69 ± 6.86	17.08 ± 3.56	-18.3	>0.05
Class IV				
FVCφ	2.32 ± 1.08	2.58 ± 0.90	+1.4	>0.05
FEV1φ	1.87 ± 1.04	2.04 ± 0.75	+9.1	>0.05
PEFR§	242.00 ± 68.25	203.50 ± 54.47	-15.9	>0.05
RVφ	1.63 ± 0.82	1.91 ± 1.23	+17.1	>0.05
TLCφ	3.87 ± 1.47	4.04 ± 1.54	+4.4	>0.05
DLCOψ	16.31 ± 3.86	16.39 ± 2.45	+0.1	>0.05

DLCO - Diffusion capacity, FVC - Forced vital capacity, FEV1 - Forced expiratory volume in 1 second, PEFR - Peak expiratory flow rate, RV - Residual volume, TLC - Total lung capacity.

φ Values are in liters

§ Values are in liters/min

ψ Values are in ml/mmHg/min.

**Table 4:** Changes in pulmonary function test at 3 months after mitral valve replacement

	Preoperative	Postoperative (3 months)	% change	*P*-value
Class II				
FVCφ	2.10 ± 0.57	2.54 ± 0.80	+20.9	>0.05
FEV1φ	1.84 ± 0.50	2.21 ± 0.91	+20.1	>0.05
PEFR§	241.38 ± 97.21	225.25 ± 80.50	-6.7	>0.05
RVφ	1.68 ± 0.68	1.49 ± 0.43	-11.3	>0.05
TLCφ	4.1 4 ± 1.31	3.94 ± 1.1 9	-4.8	>0.05
DLCOψ	19.92 ± 6.28	21. 90 ± 6.96	+9.9	>0.05
Class III				
FVCφ	2.75 ± 0.95	2.91 ± 0.88	+5.8	>0.05
FEV1φ	2.55 ± 0.84	2.69 ± 0.85	+5.5	>0.05
PEFR§	301.73 ± 89.66	31 1.73 ± 74.25	+3.3	>0.05
RVφ	1.40 ± 0.39	1.35 ± 0.27	-3.6	>0.05
TLCφ	3.75 ± 1.04	3.96 ± 0.87	+5.6	>0.05
DLCOψ	17.69 ± 6.86	2 1.67 ± 6.74	+22.5	>0.05
Class IV				
FVCφ	2.32 ± 1.08	3.07 ± 1.1 2	+32.3	<0.05
FEV1φ	1.87 ± 1.04	2.29 ± 1.09	+22.5	>0.05
PEFR§	242.00 ± 68.25	302.07 ± 112.87	+24.8	>0.05
RVφ	1.63 ± 0.82	1.70 ± 0.82	+4.3	>0.05
TLCφ	3.87 ± 1.47	4.37 ± 1.80	+12.9	>0.05
DLCOψ	16.31 ± 3.86	18.56 ± 4.56	+13.8	>0.05

DLCO - Diffusion capacity, FVC - Forced vital capacity, FEV1 - Forced expiratory volume in 1 second, PEFR - Peak expiratory flow rate, RV - Residual volume, TLC - Total lung capacity.

φ Values are in liters

§ Values are in liters/min

ψ Values are in ml/mmHg/min.

### FVC

**Figure 1:**
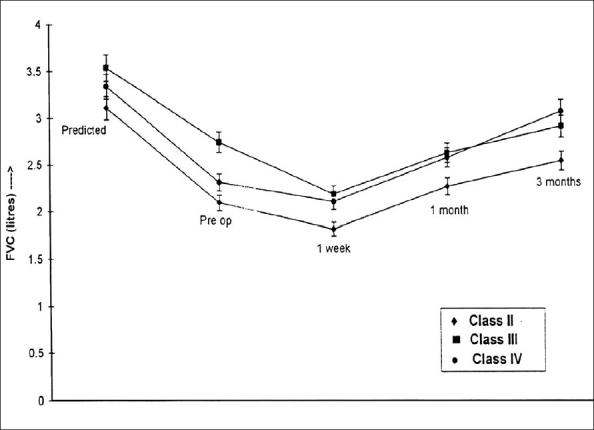
Changes in forced vital capacity (FVC) before and after mitral valve replacement

In all three classes of patients, FVC was significantly reduced in the preoperative period. There was a tendency of the values to decrease in immediate postoperative period and then improve to preoperative value [Figure 1]. However, they were not statistically significant except in NYHA Class IV, where the values were significantly improved as compared to preoperative values (3.07 ± 1.12 L at 3 months and 2.32 ± 1.08 L preoperatively, *P* < 0.05). The difference in values among the classes was not statistically significant.

### FEV1

FEV1 was significantly reduced in patients in all the three classes. In immediate postoperative period, there was a further deterioration in values of FEV1 in Class II and Class III patients. At 3 months, values were almost equal to preoperative values. There was a statistically significant variation between the groups from the preoperative values for Class II and Class III patients at 1 week only (1.84 ± 0.50 L and 2.55 ± 0.84 L preoperatively, which deteriorated to 1.26 ± 0.33 L, *P* < 0.01; and 2.11 ± 0.71 L, *P* < 0.05 at 1 week for Class II and Class III respectively).

### PEFR

PEFR values were found to be decreased in preoperative period in all three classes of patients under study. There was a statistically significant decrease in PEFR values at 1 week (241.38 ± 97.21 preoperatively to 148.88 ± 7.47, *P* < 0.01) in Class II patients. There was a significant difference from the preoperative values in the peak expiratory flow rates at 1 week between Class II (a statistically significant deterioration of 38.3%, *P* < 0.01), Class III (a fall of 2.6%, *P* > 0.05) and Class IV (a fall of 20.3%, *P* > 0.05). There was slight improvement at 1 month (-12.9%, -0.1% and -15.9% respectively for Classes II, III and IV). This was however statistically insignificant. At 3 months, Class IV patients demonstrated a marked improvement of 24.8%.

### RV

The preoperative and postoperative values of RV in all NYHA classes were not statistically significant from predicted and preoperative values respectively.[9] No inter-group variation could be established to be statistically significant.

### TLC

TLC was significantly reduced in Class III preoperatively. There was a reduction in the values of total lung capacity at 1 week in Class II (3.50 ± 1.20 L, *P* < 0.01, -15.5%) and Class III (3.22 ± 0.60 L, *P* < 0.05, -14.1%). There was no statistically significant difference among the classes.

### DLCO

**Figure 2:**
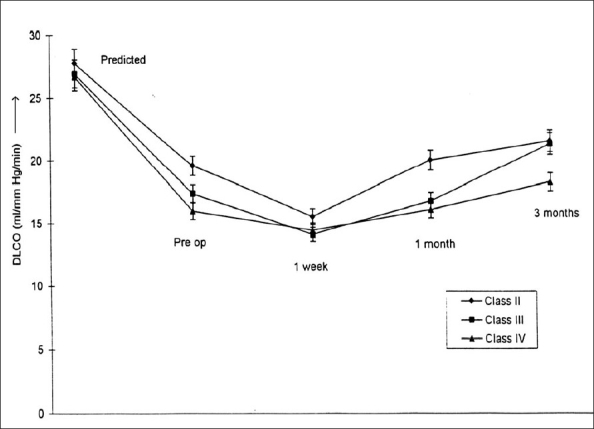
Changes in diffusion capacity (DLCO) before and after mitral valve replacement

DLCO was reduced preoperatively in patients in all the classes, and the percentage reduction seemed to correlate with the class of disease (-29.2, -35.2 and -39.7% for NYHA Class II, III and IV respectively). There was marginal improvement in the values of diffusion capacity up to 3 months of follow-up [Figure 2]. No statistically significant difference was found among the various classes.

**Figure 3:**
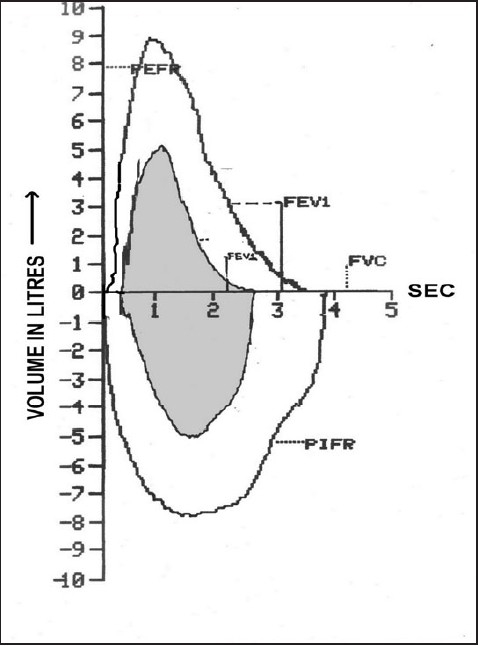
Representative flow volume loop showing gross derangement of pulmonary functions in severe mitral stenosis (shaded area). FVC - forced vital capacity, FEV1 - forced expiratory volume in 1 second, PEFR - peak expiratory flow rate, PIFR – peak inspiratory flow rate

The pattern of pulmonary functions was analyzed. Significant changes in pulmonary functions were seen preoperatively in most of the patients [Figure 3]. In Class II, only 1 patient had normal pulmonary function, whereas 4 had restrictive and 3 had moderate obstructive airway disease preoperatively. At 3 months, 50% of patients had normal pulmonary functions; whereas 2 patients had moderate restrictive disease and another 2 had moderate obstructive disease. In class III, majority of the patients (72.7%) had restrictive lung disease preoperatively. As many as 45.5% of patients continued to have restrictive defect at 3 months of follow-up, and 6 cases had normal pulmonary functions.

Out of the 6 patients in NYHA Class IV, 50% had obstructive airway disease preoperatively; whereas severe restrictive lung disease was found in 1 patient. At 3 months of follow-up, 50% of patients had normal pulmonary functions. Obstructive airway disease was present in 2 patients and 1 patient had mild restrictive pattern at 3 months after surgery.

Of the 25 patients, 13 had restrictive lung disease, 6 had obstructive airway disease and another 6 had normal pulmonary functions preoperatively. At 3 months after surgery, 9 had restrictive lung disease (an improvement in 69.2% of the 13) and 3 had obstructive airway disease (an improvement in 50% of the 6) and 13 patients had normal pulmonary functions (an overall improvement to normal in 69.2%). Of the 3 smokers in the study, 2 had moderate obstructive airway disease and 1 had normal pulmonary functions. At the end of follow-up, all three had normal pulmonary functions (66.7% improvement).

## Discussion

Several studies have found impairment of pulmonary functions in mitral valve disease. Palmer and Friedman found a decrease in pulmonary functions and stated that there was a poor correlation of pulmonary functions with severity of symptoms.[10–11] Airway obstruction further contributed to pulmonary dysfunction in these patients. Chatterji *et al.* found FVC values were reduced in direct proportion to pulmonary artery pressure, left atrial pressure and mitral valve area and gradient in ¾ groups.[12] FEV1 was uniformly reduced in all the groups and PEFR was moderately to severely reduced. Prolonged cardiopulmonary bypass over 80 min caused further decrease in FVC. After 3 months, all these parameters improved in all - above the preoperative level but remained below the predicted values.

In the present study, in Class II there was decrease in FVC, FEV1 and PEFR. In Class III FVC, FEV1, PEFR were decreased preoperatively. In Class IV there was decrease in FVC, FEV1, PEFR. These results were consistent with the results of the previous studies, which found a decrease in spirometric parameters early after surgery.[13–17]

Residual volume and functional residual capacity were found to be increased preoperatively in previous studies.[2,12] This has been explained as a consequence of pulmonary congestion, which causes trapping of air. Due to congestion and fibrosis of the lung parenchyma, there is loss of elastic recoil and the lungs fail to return to the normal resting expiratory levels. Bronchial reactivity, edema and reactive fibrosis of the small airways, hemosiderosis and frequent respiratory infections further exacerbate the problem of air trapping. This increase occurs however at the expense of inspiratory and expiratory reserve volumes so that the vital capacity and maximal voluntary ventilation are decreased. The work of ventilation is also increased due to stiffness of the lungs. However, our study did not show any significant change in RV preoperatively.

Spirometric parameters were significantly decreased preoperatively in our study, and the presence of obstructive airway disease in nonsmokers was evident.

Respiratory muscle wasting has been well documented in mitral stenosis and clearly contributes to development of restrictive lung disease and dyspnea in patients with longstanding mitral valve disease.[18–19]

DLCO is one parameter that has been consistently shown to be low in patients that require surgery.[20–22] The percentage decline in the values in the preoperative period correlated well with the functional class though the changes were statistically insignificant for Class II in this series. This decline has been attributed to the development of thickening of alveolo-capillary membrane, conclusively shown by electron microscopy, which causes impairment of gas exchange.[23] This is a structural change that marks the point of irreversibility of the disease in the lungs. The mild improvements that occur in the postoperative period are largely related to clearing of pulmonary exudates and recruitment of atelectatic areas of the lungs. More importantly, the decline in DLCO in the preoperative period also marks the beginning of a phase where restrictive lung physiology becomes predominant. In contrast to this, the early stages of dysfunction are of obstructive lung physiology, which is to a large extent reversible with reduction of left-sided pressures.

The genesis of dyspnea in mitral valve disease is multifactorial. High left atrial pressures and pulmonary hypertension are mainly responsible.[22,24] It has however eluded most studies to show a consistent correlation and working formulation between LA pressures, level of pulmonary hypertension, changes in PFT and the clinical class of dyspnea. The progression of dyspnea, vis-à-vis the aforementioned factors, in spite of the ability to objectively and accurately measure them, remains unpredictable. Patients with similar lesions and PFT profiles may still present as different classes of dyspnea. There was a poor correlation between pulmonary functions and severity of symptoms among the various functional classes in the present study. Different studies could correlate pulmonary functions and the functional class.[12,14,15]

Musthafa found an initial decrease in pulmonary functions but an improvement in late postoperative period except in most severe cases.[16] Chandra and co-workers found that there was an overall improvement in spirometric parameters at 3 months of follow-up after valve replacement, although the values remained lower than the predicted.[14] Ghosh *et al.* have reported a significant decrease in FVC, FEV1, flow rates at 25–75% of expired vital capacity (FEF 25–75%) and maximum voluntary ventilation (MVV) in all patients at discharge.[15] In our experience, after 3 months all these parameters improved in all above the preoperative level but remained below the predicted values. Despite improvement in NYHA class, impaired spirometry was observed in 11/31 patients. Functional or hemodynamic improvement did not correlate with spirometric changes. This early decline was also seen in our study. Majority of the patients in our study who were in NYHA Class III and Class IV were ventilated overnight. All patients had a median sternotomy, and pleura was not breached in any of our cases. Adequate analgesia in the form of opiates was administered in the postoperative period, and aggressive chest physiotherapy was started. The patients were made ambulatory at the earliest. None of these measures affected the decline in PFT at 1 week in our series.

Kadam *et al.* ascribe this early deterioration to residual healing process and thoracotomy pain in their closed valvotomy patients.[25] None of our patients had a thoracotomy, and possibly this early dysfunction is related more to the effects of cardiopulmonary bypass. However, these changes are seen even after successful percutaneous mitral valvotomy.[21] There was a tendency of pulmonary functions to reach preoperative values at 3 months, but a statistically significant improvement could not be demonstrated in most of the parameters.

Seboldt and Singh found that changes in pulmonary functions were irreversible.[17,26] Rhodes found changes in spirometric parameters and lung volumes to be reversible.[27] We could find a statistically significant improvement in the value of only FVC in Class IV patients at 3 months after surgery. Most of the restrictive parameters do not show any significant change in the long term after surgery.

It is evident that in the course of the disease, a point of irreversibility is reached, beyond which valve replacement does not improve the pulmonary functions to normal levels. However, it is also evident that even when the treatment is offered beyond this point, most patients, if not all, are likely to improve their class of dyspnea, in spite of failure to objectively improve most parameters of their pulmonary functions. Unlike in congenital heart diseases with pulmonary hypertension, this point of irreversibility does not make the patient unsuitable for a valve replacement. Treatment therefore at any point in the disease symptomatically improves the patient. However, it may be important to intervene early, even when the symptoms are minimal, to prevent irreversible changes in the pulmonary functions.[28]

we conclude from the present study that lung volumes and diffusion capacity decrease in majority of the patients with rheumatic mitral valve disease. There is a poor correlation between the degree of change in pulmonary functions and functional class. The pulmonary functions deteriorate immediately after surgery and then recover gradually over a period of 3 months; however, they remain below the predicted values. Clinical improvement does not correlate with improvements in objective pulmonary function parameters. It needs long-term follow-up to assess the complete changes in pulmonary function tests after mitral valve replacement.

### Limitations

The study was performed in a small group of patients[25] with short duration of follow-up. Further, the study group was limited to rheumatic mitral disease. There were no patients with mild mitral valve disease. This group would probably be the most interesting group to study in terms of preoperative changes and reversibility following valve replacement. Evaluation of exercise-induced changes in pulmonary functions would have provided a greater insight into the pulmonary mechanics and a better representation of the actual clinical responses.
